# Gamma-glutamyltransferase as novel biomarker in patients with uterine leiomyosarcoma

**DOI:** 10.1038/srep33757

**Published:** 2016-09-20

**Authors:** Richard Schwameis, Christoph Grimm, Thomas Brodowicz, Edgar Petru, Katrin Hefler-Frischmuth, Christine Staudigl, Alexander Reinthaller, Georg Heinze, Stephan Polterauer, Mariella Polterauer

**Affiliations:** 1 Department of General Gynaecology and Gynaecological Oncology, Gynecologic Cancer Unit - Comprehensive Cancer Center, Medical University of Vienna, Vienna,1090, Austria; 2 Clinical Division of Oncology, Department of Medicine 1, Comprehensive Cancer Center - Medical University Vienna, Vienna, 1090, Austria; 3 Department of Obstetrics and Gynaecology of the Medical University of Graz, Graz, 8036, Austria; 4 Department of Internal Medicine I, Barmherzige Schwestern Hospital Linz, Linz, 4020, Austria; 5 Department of Gynaecology, Barmherzige Schwestern Hospital Linz, Linz, 4020, Austria; 6 Karl Landsteiner Institute for General Gynecology and Experimental Gynecologic Oncology, Vienna,1090, Austria; 7 Center for Medical Statistics, Informatics and Intelligent Systems, Medical University of Vienna, Vienna, 1090, Austria

## Abstract

Gamma-glutamyltransferase (GGT) is an established marker for proliferative/apoptotic balance and has been associated with cancer risk and prognosis. The aim of this study was to evaluate the value of pre-treatment GGT serum levels as prognostic biomarker in patients with primary uterine leiomyosarcoma (ULMS). Data of women with ULMS were extracted from a multi-center database. Pre-treatment GGT serum levels were measured and patients assigned to predefined GGT risk groups. GGT values were correlated with clinico-pathological parameters and univariate and multivariable survival analyses were performed. A total of 44 patients with ULMS were analyzed. Mean (SD) pre-therapeutic GGT serum level was 33.8 (39.8) U/L. In Figo Stage I versus II-IV mean (SD) GGT values were 28.8 (34.0) U/l and 43.5 (49.2) U/l, respectively (p = 0.25). Five-year overall survival (OS) rates in ULMS patients with normal low versus higher GGT levels were 70% and 37%, respectively (*p* = 0.043). Univariate and multivariable analyses revealed that higher GGT serum levels (*p* = 0.043, *p* = 0.005) and high histological grade (*p* = 0.029, *p* = 0.012) were independently associated with impaired OS, respectively. Higher pre-treatment GGT serum levels were independently associated with unfavorable prognosis in women with ULMS. Thus, GGT seems to be a useful novel biomarker in ULMS.

Gamma-glutamyltransferase (GGT) is a membrane bound enzyme essential in the glutathione (GSH) metabolism that protects cells from reactive oxygen species^1^. GGT is an established biomarker for apoptotic balance and promotes carcinogenesis, malignant transformation, and tumor progression^2^,^3^. It has been described that pathologic states of oxidative stress, as found within carcinogenesis increase GSH and GGT levels^1^,^4^.

Serum GGT was shown to be associated with an increased risk of cancer development^5^,^6^ and is elevated in a variety of solid tumors^7^. Furthermore, GSH may play a relevant role in the development of chemo-resistance in malignancies including soft tissue sarcoma^8^. Recent studies investigated the prognostic role of serum GGT and GGT expression in various gynecologic malignancies including endometrial, cervical, and ovarian cancer^9^,^10^,^11^.

ULMS can be considered as an orphan disease with distinctive clinical differences to non-uterine leiomyosarcoma and reports on valid pre-therapeutic prognostic biomarkers are limited^12^. The aim of the present multicenter study was to investigate the influence of pre-therapeutic GGT on survival of patients with ULMS.

## Results

Patients’ characteristics are provided in Table 1. Mean (SD) pre-therapeutic GGT serum levels in patients with ULMS were 33.8 (39.8) U/L. Patients were assigned to previously defined GGT groups^10^,^13^. We observed a non-significant trend that higher pre-therapeutic GGT serum levels were associated with more advanced tumor stage and large tumor volume, when compared to patients with normal-low GGT (Table 2).

**Figure 1:**
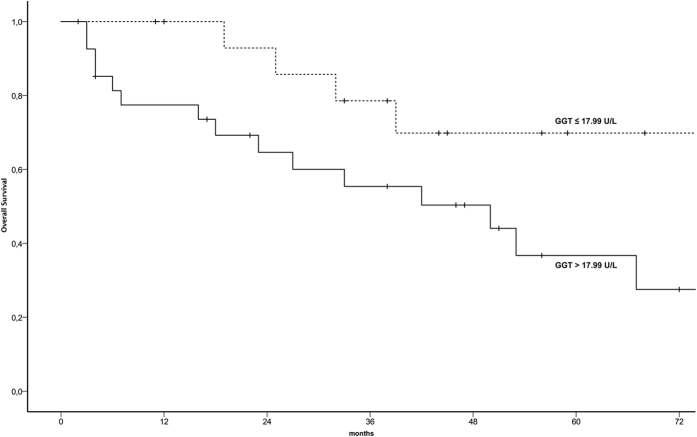
Overall survival in patients with uterine leiomyosarcoma according to pre-therapeutic GGT serum levels. GGT: Gamma-glutamyltransferase.

In univariate and multivariable survival analysis GGT serum levels, patients’ age, tumor stage, and histological grading were analyzed as prognostic parameters for OS. Table 3 provides results of the univariate Kaplan-Meier analysis and the multivariable Cox regression model. Women with higher pre-treatment GGT serum levels showed impaired OS compared to women with normal low levels (<17.99 U/l) and 5-year OS rates of 37% and 70% were observed, respectively (*p* = 0.043). Kaplan-Meier survival curves, showing the association between pre-therapeutic GGT serum groups and OS are shown in Fig. 1.

In a sensitivity analysis we dichotomized GGT at the 25^th^ percentile value of 17.5, which resulted in an adjusted hazard ratio of 2.77 (95% CI 0.93–10.8, p = 0.068). In multivariable analysis higher GGT serum levels, advanced patients’ age and high-grade histology were associated with unfavorable prognosis. Using age as a continuous variable in the Cox model resulted in a hazard ratio for age (per decade) of 0.62 (95% CI 0.33–1.24, p = 0.168), but did not materially change the hazard ratio for GGT (HR 1.80 per each doubling of GGT, 95% CI 1.19–2.68, p = 0.006).

Interestingly, in our cohort 29 patients had FIGO stage I ULMS. In eight of these patients cancer-related death was observed and seven (87.5%) of these patients had GGT serum levels of ≥17.99 U/L. A total of 10 (47.6%) of the remaining 21 patients with FIGO stage I were still alive within the observation period had GGT serum levels of ≥17.99 U/L. With respect to tumor grade all 8 patients that died from FIGO I ULMS revealed grade 3 histology. Within this cohort, 4 and 3 patients had a maximal tumor size ≤5 cm and >5 cm, respectively. The tumor size of one patient was not exactly documented. Out of 4 patients with a tumour size ≤5 cm and fatal outcome 3 (75%) patients had high serum GGT levels (≥17.99 U/L).

## Discussion

The present study shows that pre-therapeutic serum GGT is a novel prognostic biomarker for patients with ULMS, with higher levels being associated with worse outcomes. Multivariable analysis revealed prognostic relevance of GGT independent of established parameters such as age, FIGO stage, and tumor size. Results of previous studies indicate that GGT reflects a valid prognostic biomarker for different solid tumors, including gynecologic malignancies but no data for ULMS have been published so far^9^,^10^,^14^. From a clinical view it seems plausible that GGT, a marker of oxidative stress, provides prognostic information. By revealing the amount of pathologic oxidative stress in cancer patients, GGT most likely reflects the extent of malignant cell transformation and cell turnover caused not only by the extent of tumor load, but also by the tumor’s aggressiveness, and biological behavior^11^. In our study, GGT serum levels were only slightly (non-significantly) higher in patients with large tumors (>10 cm) and advanced stage (FIGO II-IV). On the other hand, 7 out of 8 patients that died from early stage disease had elevated GGT serum levels. In the past years, several studies investigated the biology of GGT and its’ impact on cancer development, progression, and drug resistance^15^. GGT and GSH play an important role in the cellular antioxidant defense mechanism by binding reactive oxygen species^1^,^16^. Interestingly, several studies showed that GGT influences the cellular proliferative-apoptotic balance by exerting pro-oxidant effects at the membrane surface level and in the extracellular microenvironment^7^,^15^. By providing cysteine, GSH has been linked with rapid tumor growth and cancer survival^2^. Since expression of GGT provides a growth advantage for the tumor tissue, it seems reasonable that elevated GGT serum levels are associated with rapidly growing and large tumors. This would explain that patients with small tumors but high GGT serum levels, as seen in this cohort, were associated with poor prognosis.

Enhancing the capacity of GSH and its associated enzymes, in order to protect cells from redox-related changes or environmental toxins, represents a persistent aim in the search for cyto-protective strategies against cancer. Novel compounds that act as uncompetitive inhibitors of human GGT with low toxicity and a large therapeutic window were already tested in preclinical models. New potent agents are currently in the process of further development with GGT as a potential therapeutic target^17^.

Due to the low prevalence of the disease, patients with ULMS are often studied together with non-uterine LMS or soft tissue sarcoma (STS) patients^12^. Glutathione concentration and GGT activity were previously investigated in extremity STS^8^. It was found that glutathione and GGT were significantly elevated in patients with aggressive disease, especially in high-grade and metastatic STS. In addition, increased glutathione levels were found in pretreated tumors previously exposed to doxorubicin-based chemotherapy. Therefore it was postulated that glutathione might play a role in the development of resistance to chemotherapeutic agents in STS that might subsequently influence patients’ prognosis. The mechanism underlying the correlation between expression of GGT and drug resistance seems to be the ability of GGT to cleave extracellular GSH and thereby, provide cells with an additional source of cysteine with which to increase intracellular GSH levels^18^.

Owing to the retrospective design of this study several limitations have to be taken into account. Firstly, this retrospective study recruited patients from a long treatment period of more than 15 years. In this period of time therapeutic regimes for patients with ULMS have changed profoundly and the influence of this change could not be reflected in this trial. Secondly, the number of patients included into this study is relatively small. On the other hand, ULMS is an orphan disease for which only few prognostic parameters are established yet. This is the first study that evaluated pre-therapeutic serum GGT levels as a potential prognostic parameter in this population. Therefore, we think that these findings might be interesting from a clinical point of view and seem biologically plausible. Undoubtedly, the results found retrospectively need to be confirmed in future studies. At present, GGT is widely used in clinical routine mainly to monitor liver function and is a cost-effective and readily available prognostic parameter. Especially due to the lack of reliable prognostic serum biomarkers for women diagnosed with ULMS, serum GGT levels might be clinically useful, when included in future trials.

An interesting aspect is the potential role of GGT serum levels for screening purposes that has been investigated in several recent studies. The population-based AMORIS study showed an association between elevated serum GGT levels and overall cancer risk^19^. A similar study showed an association between elevated GGT serum levels and increased cervical cancer risk^6^. However, in both studies no GGT treshold for a population based screening has been established^6^,^19^. ULMS is considered an orphan disease, therefore population based screening for ULMS might not be reasonable. However, in clinical practice patients with rapidly growing uterine myoma, or post-menopausal women with growing myoma are often suspected to have underlying malignancies of the uterus in clinical practice. One might argue that in these patients GGT measurements could be useful to differentiate between myoma and ULMS. However, to the best of our knowledge there are no data supporting this strategy and future studies are necessary to investigate the potential role of GGT for differential diagnosis in this cohort.

In summary, we identified GGT as promising independent prognostic biomarker in women with ULMS. If the current results can be validated in an independent prospective trial, it might be reasonable to divide ULMS patients into subgroups according to GGT-levels to facilitate patient counseling and treatment surveillance. Until then, our results may be considered when planning future clinical trials.

Further research is needed to address the biological mechanisms linking GGT to ULMS progression and prognosis.

## Materials and Methods

### Patients

A total of 44 consecutive patients diagnosed with ULMS were treated between 1996 and 2014 at the Comprehensive Cancer Center Vienna, Vienna, Department of Obstetrics and Gynecology of the Medical University of Graz, and the Department of Gynecology, Barmherzige Schwestern Hospital Linz, Austria and enrolled in the present study. Clinical data were obtained using available tumor databases and by electronic chart review. The 2009 International Federation of Gynecology and Obstetrics (FIGO) classification system was used^20^. Primary tumor assessment was performed by clinical examination, transvaginal sonography and in some cases with magnetic resonance imaging (MRI) and/or computed tomography (CT). Treatment consisted of surgery including hysterectomy and bilateral salpingo-oophorectomy. Pelvic and/or paraaortic lymphadenectomy was carried out in the case of intraoperative palpably enlarged lymph nodes. Surgical cytoreduction was performed in women with extrauterine disease as described previously^12^. If clinically indicated, radiation therapy, adjuvant and/or palliative chemotherapy was given based on the physicians’ choice. In all patients included in this study a pre-therapeutic physical examination by a specialist in internal medicine was performed in order to rule out the presence of liver diseases. Patients with liver diseases or liver metastasis were not eligible for analysis. All patients were included into the institutions’ follow-up care program. The latter included clinical examination. Patients were seen every three to four months for the first three years, every six months for the following two years and afterwards annually up to ten years. If recurrent disease was suspected imaging methods based on the physicians choice were conducted.

### Ethics

The study was approved by the Ethics Committee of the Medical University of Vienna (IRB approval number: 1520/2012). All patients consented to treatment according to institutional guidelines, and all patients had consented to anonymized assessments and analysis of data and outcome of therapy. Since this study was a retrospective analysis the ethics committee waived the requirement to obtain distinct informed consent from patients. All patient records were anonymized and de-identified prior to analysis. This study was performed in accordance to the ICH Harmonized Tripartite Guideline for Good Clinical Practice, the Declaration of Helsinki and the guidelines of the Ethics Committee of the Medical University of Vienna.

### GGT Measurement

As part of clinical routine, blood samples for evaluation of serum GGT levels were obtained by peripheral venous puncture 24–48 hours prior to primary surgery. GGT concentrations were analyzed with an enzyme kinetic assay (Modular Hitachi 747 and Hitachi 917, Roche Diagnostics), as described previously^21^. Patients were assigned to the previously described GGT groups as follows: GGT < 17.99 U/L: group A (normal low), 18.00 to 35.99 U/L: group B (normal high), 36.00 to 71.99 U/L: group C (elevated), and >72.00 U/L: group D (highly elevated)^10^,^13^.

### Statistical Analysis

Values are given as mean (standard deviation [SD]). Students’ T- tests and one-way ANOVA tests were applied to compare mean GGT serum levels and clinico-pathological findings. P-values of <0.05 were considered statistically significant. Survival probabilities were calculated by the product limit method of Kaplan and Meier. Differences between groups were tested using the log-rank test. The results were analyzed for the endpoint of overall survival (OS). Survival times of patients that were still alive at the last follow up visit were censored with the last follow-up date. Univariate survival analysis was performed using log-rank test and Cox Regression analysis. Kaplan-Meier survival curves were computed. Multivariable analysis was conducted using Cox regression including as independent variables log2-transformed GGT and the clinically relevant variables age (dichotomized at the median value of 55.3), tumor stage (FIGO II-IV *vs*. FIGO I), and grading (G2-G3 *vs*. G1). Because there were no events in the G1 group, ordinary maximum likelihood estimation could not be performed and was replaced by the penalized likelihood method recommended for such situations by^22^. Statistical analysis was performed using the commercially available statistical software SPSS 23.0 for MAC (SPSS 23.0, IBM Inc., Armonk, NY). All analyzed data are available as Supplementary Database 1.

## Additional Information

**How to cite this article**: Schwameis, R. *et al*. Gamma-glutamyltransferase as novel biomarker in patients with uterine leiomyosarcoma. *Sci. Rep*. **6**, 33757; doi: 10.1038/srep33757 (2016).

## Supplementary Material

Supplementary Information

## Figures and Tables

**Table 1:** Demographic characteristics of 44 patients with uterine leiomyosarcoma.

Variable	N (%) or Mean (SD)
Patients enrolled	44
Age at diagnosis (years)	55.3 (10.5)
Pretherapeutic GGT (U/L)	33.8 (39.8)
Tumorstage	
FIGO I	29 (65.9)
FIGO II	2 (4.5)
FIGO III	1 (2.3)
FIGO IV	12 (27.3)
Tumorsize (cm)	
<5	11 (25)
5–10	16 (36.4)
>10	13 (29.5)
Unknown	4 (9.1)
Histological Grading	
G1	6 (13.6)
G2	4 (9.1)
G3	27 (61.4)
Unknown	7 (15.9)
Primary Metastastatic Site	
Lymph nodes	2 (4.5)
Lungs	11 (25.0)
Bone	2 (4.5)
Others	7 (15.9)
Status at last follow up	
Alive	25 (56.8)
Dead	19 (43.2)
Follow Up Time (months)	57 (33–89)*

SD: standard deviation, GGT: Gamma-glutamyltransferase, FIGO: International Federation of Gynaecology and Obstetrics.

^*^Given as median (25^th^–75^th^ percentiles).

**Table 2:** Mean Gamma-glutamyltransferase values categorized by clinico-pathological parameters.

Parameter	Mean GGT U/l (SD)	*p*-value
Age (years)		0.35^a^
<55.3	28.6 (23.9)	
>55.3	40.0 (53.2)	
Tumorstage		0.25^a^
FIGO I	28.8 (34.0)	
FIGO II-IV	43.5 (49.2)	
Tumorsize (cm)		0.34^b^
<5	26.2 (21.3)	
5–10	27.7 (17.0)	
>10	47.1 (64.8)	
Grading		0.78^a^
G1	31.3 (29.1)	
G2-G3	36.8 (45.2)	

SD: standard deviation, GGT: Gamma-glutamyltransferase, FIGO: International Federation of Gynaecology and Obstetrics.

^a^*p*-value was calculated with students’ t-test.

^b^*p*-value was calculated with One-way ANOVA.

**Table 3:** Univariate and multivariable survival analysis of 44 patients with uterine leiomyosarcoma.

Parameter	Univariate		Multivariable	
	5-year OS	*p*-value	HR (95% CI)	*p*-value
GGT		0.043	1.90 (1.22–2.96)*	0.005
≤17.99 U/L	69.8%			
>17.99 U/L	36.7%			
Age		0.084		
<55.3	32.2%		1 (ref.)	
>55.3	68.5%		0.27 (0.07–0.83)	0.022
Tumorstage		0.003	2.38 (0.88–6.36)	0.085
FIGO I	67.1%		1 (ref.)	
FIGO II-IV	20.0%		1.29 (0.44–3.81)	0.636
Grading		0.029		
G1	100%		1 (ref.)	
G2-G3	34.8%		13.8 (1.59–1812)	0.012

GGT: Gamma-glutamyltransferase.

OS: overall survival, HR: hazard ratio, CI: confidence interval, ref. : reference level.

^*^Per each doubling of GGT, FIGO: International Federation of Gynaecology and Obsterics.
